# Transcriptional Factors Mediating Retinoic Acid Signals in the Control of Energy Metabolism

**DOI:** 10.3390/ijms160614210

**Published:** 2015-06-23

**Authors:** Rui Zhang, Yueqiao Wang, Rui Li, Guoxun Chen

**Affiliations:** 1 State Food and Drug Administration Hubei Center for Medical Equipment Quality Supervision and Testing, 666 High-Tech Avenue, Wuhan 430000, China; E-Mail: lizzyzr@gmail.com; 2 Department of Nutrition and Food Hygiene, Wuhan University, 185 East Lake Road, Wuhan 430071, China; E-Mail: elle529@126.com; 3 Department of Nutrition, University of Tennessee at Knoxville, 1215 West Cumberland Avenue, Knoxville, TN 37996, USA; E-Mail: gchen6@utk.edu

**Keywords:** vitamin A, retinoic acid, retinoic acid receptor, retinoid X receptor, hepatic nuclear factor 4α, chicken ovalbumin upstream-transcription factor II, peroxisome proliferator activated receptor β/δ, metabolism

## Abstract

Retinoic acid (RA), an active metabolite of vitamin A (VA), is important for many physiological processes including energy metabolism. This is mainly achieved through RA-regulated gene expression in metabolically active cells. RA regulates gene expression mainly through the activation of two subfamilies in the nuclear receptor superfamily, retinoic acid receptors (RARs) and retinoid X receptors (RXRs). RAR/RXR heterodimers or RXR/RXR homodimers bind to RA response element in the promoters of RA target genes and regulate their expressions upon ligand binding. The development of metabolic diseases such as obesity and type 2 diabetes is often associated with profound changes in the expressions of genes involved in glucose and lipid metabolism in metabolically active cells. RA regulates some of these gene expressions. Recently, *in vivo* and *in vitro* studies have demonstrated that status and metabolism of VA regulate macronutrient metabolism. Some studies have shown that, in addition to RARs and RXRs, hepatocyte nuclear factor 4α, chicken ovalbumin upstream promoter-transcription factor II, and peroxisome proliferator activated receptor β/δ may function as transcriptional factors mediating RA response. Herein, we summarize current progresses regarding the VA metabolism and the role of nuclear receptors in mediating RA signals, with an emphasis on their implication in energy metabolism.

## 1. Introduction

Vitamin A (VA, retinol) is a lipophilic micronutrient. Its derivatives and compounds that possess similar or equivalent activities are generally referred to as retinoids, which have been shown to play a role in a large spectrum of mammalian physiological processes such as spermatogenesis, fertilization, pregnancy maintenance, morphogenesis, organogenesis, and fetal and perinatal growth [1]. In adults, retinoids regulate reproduction, immunity, vision, and metabolism, and maintain proper functions of skin, lung, bone marrow, liver, and neuronal system. Among retinoids, retinal plays a key role in vision and retinoic acid (RA) has both genomic and non-genomic regulatory functions [2,3].

The majority of VA’s physiological functions are mediated by RA, which exists in multiple isomeric forms and regulates gene expression. In this process, RA functions as ligand for transcriptional factors associated with the RA response elements (RAREs) located in the promoters of RA-responsive genes [4]. The most studied transcriptional factors bound to RAREs are RA receptors (RARs) and retinoid X receptors (RXRs), which are members of the nuclear receptor (NR) superfamily. Recently, experimental results have shown that other members of the NR superfamily are capable of mediating RA responses in regulation of the expression of genes involved in energy metabolism. Therefore, the aim of this review is to summarize the current progresses and propose potential mechanisms by which these NRs take part in the RA signaling events.

## 2. Uptake and Metabolism of Vitamin A (VA)

**Figure 1:**
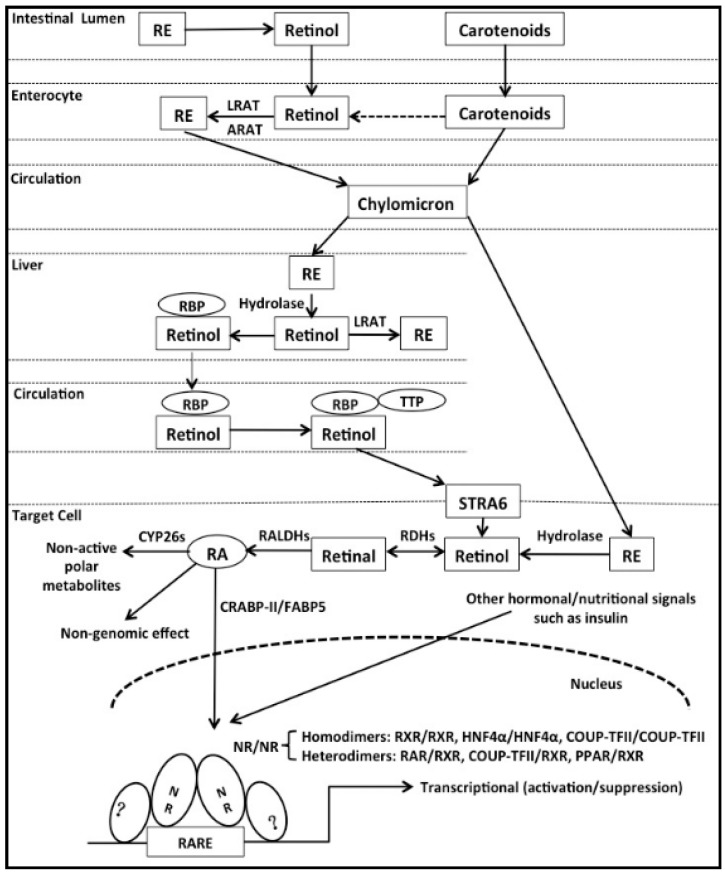
Metabolism of vitamin A (VA) and retinoic acid (RA) signaling. In the small intestine, retinyl esters (REs) are metabolized to retinol, which is converted to REs in enterocytes. REs and carotenoids are then incorporated into chylomicrons and exported into the circulation. The chylomicron remnants are taken up by the hepatocytes and REs are converted to retinol, which is either converted to REs for storage in stellate cells or bound to retinol-binding protein (RBP) for release into the circulation. In the bloodstream, holo-RBP associates with transthyretin (TTR) to form a holo-RBP-TTR complex to avoid elimination by the kidney and to ensure delivery to target tissues via the stimulated by RA gene 6 (STRA6). A fraction of REs is also delivered by chylomicrons to extrahepatic tissues such as kidney and lung. In the cytosol of target cells, retinol is reversibly oxidized to retinal by two types of molecules with retinol dehydrogenase (RDH) activity: alcohol dehydrogenases (ADHs) and short-chain dehydrogenases (SDRs). Retinal is then irreversibly oxidized to RA by retinal dehydrogenases (RALDHs). The synthesized RA, together with RA directly taken up from the extracellular matrix, where it may have been released by other cells, can be degraded to non-active polar metabolites by enzymes of the cytochrome P450 enzyme (CYP26) family. In addition to non-genomic effect, RA can also be shuttled to the nucleus by cellular RA binding protein II (CRABP-II) and fatty acid binding protein 5 (FABP5) and binds to nuclear receptors (NRs). In the nucleus, retinoid X receptor (RXR), hepatic nuclear factor 4α (HNF4α), and chicken ovalbumin upstream promoter-transcriptional factor II (COUP-TFII) can form homodimers, while RA receptor (RAR), peroxisome proliferator activated receptor β/δ (PPARβ/δ), and COUP-TFII can form heterodimers with RXR. These dimers bind to RA response elements (RAREs) located in the promoters of RA target genes. Binding of RA to these NRs modulates expressions of target genes. Hormonal and nutritional signals such as insulin may also affect the binding of NRs to RAREs. In the case of hepatic glucokinase gene (*Gck*), additional unknown co-factors (as indicated by “?”) bound to the promoter region proximal to the RARE may contribute to the transcriptional regulation.

For humans, dietary molecules with VA activities include preformed VA (retinol and retinyl esters, REs) and provitamin A carotenoids [5], which are derived from animal and plant sources, respectively. Fifty out of more than 600 carotenoids identified from plants serve as precursors of VA in humans [5]. Animal products such as eggs and liver contain VA predominantly in the form of REs. As shown in Figure 1, in the intestinal lumen, REs from the diet are hydrolyzed into retinol and fatty acids by several enzymes, including pancreatic lipase, pancreatic carboxyl ester lipase, and one or more RE hydrolases associated with the brush border membranes [6]. The uptake of retinol and carotenoids by enterocytes of the small intestine is the first step in their utilization by the organism. In enterocytes, part of the carotenoids can be converted to retinal by β-carotene-15,15′-monooxygenase (central cleavage), and retinal is then reduced to retinol by aldehyde reductase [7]. Another enzyme, β,β-carotene-9′, 10′-dioxygenase (eccentric cleavage), may also contribute to the conversion of carotenoids to retinal [8].

Within the enterocytes, retinol is re-esterified with long-chain fatty acids through the actions of two enzymes: lecithin: retinol acyltransferase (LRAT) that interacts directly with the retinol-cellular retinol binding protein II (CRBP-II) complex and acyl CoA: retinol acyltransferase (ARAT) that interacts with free retinol [9,10]. It has been suggested that LRAT plays a major role under normal physiological conditions when intracellular CRBP-II level exceeds retinol level, and ARAT esterifies excess retinol when large amount of retinol is absorbed and CRBP-II is saturated [11]. Recently, it has been shown that LRAT is responsible for the preponderance of RE synthesis in the body, aside from in the intestine and adipose tissue [12]. LRAT has been cloned and identified as a integral membrane protein [13], whereas attempts to purify and clone ARAT were unsuccessful. However, recent *in vitro* and *in vivo* studies have shown that diacylglycerol acyltransferase 1 (DGAT1) can catalyze ARAT reaction [12,14]. It is also reported that monoacylglycerol 3 (MGAT3) is capable of carrying out the ARAT reaction *in vitro* [15].

In the postprandial period, REs and intact carotenoids are secreted into lymph as chylomicrons, while a minor fraction of free retinol is secreted into portal circulation [16], possibly due to the secretion of high-density lipoprotein by intestine via an ATP-binding cassette transporter ABCA1-dependent pathway [17]. In the general blood circulation, most chylomicron REs are associated with the particle during the conversion to chylomicron remnants. As a result, the majority of REs in the chylomicron remnants are taken up by hepatocytes and approximately 25% by kidney, spleen, heart, adipose tissue, lung, skeletal muscle, and adrenals [18]. Carotenoids associated with lipoproteins in chylomicron can be taken up by lipoprotein-specific receptors and converted to retinal by β-carotene-15,15′-monooxygenase [19].

In hepatocytes, REs are hydrolyzed to retinol by bile salt-independent hydrolase at the plasma membrane and by acid hydrolase in the early endosomes [20]. Un-esterified retinol may be secreted into the circulation bound to serum retinol-binding protein (RBP). In the bloodstream, retinol-bound RBP (holo-RBP) associates with another plasma protein, transthyretin (TTR), to form a holo-RBP-TTR complex which circulates at approximately 1:1 molar ratio under normal physiological conditions [21,22]. Formation of this complex can prevent the loss of small proteins like RBP by filtration in the kidney [23]. It has been thought that excess retinol is transferred to stellate cells and re-esterified into REs for storage by LRAT or ARAT [24]. However, recent studies have shown that mice lacking the LRAT gene do not have REs in their liver [25].

The uptake of plasma retinol by peripheral cells has been an area of interest. It has been shown that stimulated by RA gene 6 (STRA6), a member of a large group of “stimulated by RA” genes encoding transmembrane proteins and proteins with unknown functions, acts as a high-affinity cell-surface receptor for RBP and mediates the uptake of retinol from RBP by peripheral cells [26,27]. On the other hand, STRA6 functions to secrete retinol from cells [28]. It was recently reported that STRA6 also functions as a surface signaling receptor that activates Janus kinase (JAK)/signal transducer and activator of transcription (STAT) signaling cascade to up-regulate the expression of STAT target gene and therefore modulates insulin signaling and lipid metabolism [29,30]. In addition, reduced expression of *Stra6* in adipocytes leads to lean phenotype and improved insulin sensitivity in chow-fed mice [31]. However, deletion of *Stra6* in mice does not impair physiological functions and retinoid content in tissues excluding retinal pigment epithelium in the eye, possibly due to the functional compensation of a protein that has yet to be identified [32].

In the target cells, retinol is reversibly oxidized to retinal by two types of molecules with retinol dehydrogenase (RDH) activity: cytosolic medium-chain alcohol dehydrogenases (ADHs) and membrane-bound short-chain dehydrogenases/reductase (SDRs) [33]. There are seven ADH forms and about seventy distinct SDR forms in human [34,35]. Retinal can be then irreversibly oxidized to RA, the biologically active metabolite of VA, by retinal dehydrogenases (RALDH1, 2, 3 and 4) [36]. Importantly, RALDH1 appears to be the predominant enzyme for RA biosynthesis [37], and elevated RA is observed to control its biosynthesis by down-regulating RALDH1 through modulation of RARα and CCAAT/enhancer-binding protein β (C/EBPβ) [38,39]. Previously, it has been considered that oxidation conversion of retinol occurs in an unregulated fashion mediated by ADHs. However, recent studies have shown that RDH10 plays a critical role in controlling the oxidation of retinol [40].

Due to the potency of RA in activating expression of genes involved in a variety of physiological processes, its level should be delicately regulated. It is believed that the catabolism of RA is critical to the maintenance of RA levels in cells and tissues. In vertebrates, three cytochrome P450 enzymes (CYP26s), designated CYP26A1, CYP26B1, and CYP26C1, are able to metabolize RA to polar metabolites, such as retinoyl β-glucuronide, 5,6-epoxy-RA, 4-hydroxy-RA, 4-oxo-RA, and 3,4-didehydro-RA [36]. These enzymes differ in their expression patterns, suggesting their distinct roles in the catabolism of RA in different tissues [41]. In addition, RA can induce the mRNA expression of *Cyp26a1* and *Cyp26c1*, indicating a mechanism by which CYP26s sense the concentration of RA and regulate its catabolism accordingly [23]. In addition to its non-genomic effect in the cytosol, RA can also be directed by cellular RA binding protein II (CRABP-II) and fatty acid binding protein type 5 (FABP5) to the nucleus for activation of specific NRs [42].

## 3. Characteristics of a Retinoic Acid Response Element (RARE)

The first natural RARE was identified in the *Rarb* promoter in 1990 [43,44,45]. After deletion analysis of the *Rarb* promoter and co-transfection experiments, the sequence of a genomic fragment in the mouse mammary tumor virus promoter was located closely to the transcription initiation site and conferred RA responsiveness in monkey CV-1 kidney cells and mouse F9 teratocarcinoma cells [43]. This element was then termed RARE, and its binding to RAR is independent of the presence of RA, as demonstrated by electrophoretic mobility shift (EMSA) assays [45]. This RARE consists of a direct repeat (DR) of two motif (G/AGTTCA) separated by 5 bp, which is called DR5 element. Mutation studies demonstrated that both these motifs are necessary for RA responsiveness [43]. Soon after, other RAREs with different nucleotides spacing were identified in a number of gene promoters. Recently, genome wide chromatin immunoprecipitation followed by sequencing (ChIP-seq) has facilitated the discovery of some unexpected RAREs [46].

Most of the RAREs are composed of two hexameric motifs, 5′-(A/G)G(G/T)TCA-3′, arranged as palindromes, DRs, or inverted repeats (IRs) [47]. The most frequent DRs with 1, 2, or 5 nucleotides spacing are termed DR1, DR2, and DR5 elements, respectively. However, *in vitro* studies have shown that a significant number of RAR/RXR heterodimer-occupied sites in embryoid bodies or F9 embryonal carcinoma cells have divergent, non-canonical half-site nucleotides spacing, including DR0, DR8, and IR0 elements [48]. RAR/RXR heterodimers bind to a RARE with specific polarities. For example, DR1 elements exhibit different polarity of binding of the liganded RAR subunit compared to the DR2 and DR5 elements. For DR1 elements, the 5′ half site is recognized by a RAR and this type of RARE-bound complex acts as a transcriptional repressor. RXRs can also bind as homodimers to DR1 elements and respond to 9-*cis* RA. In contrast, for DR2 and DR5 elements, a RAR occupies the 3′ halves of these RAREs and the RARE-bound complex functions as transcriptional activator [49,50,51]. Additional arrangement of two or three hexameric motifs with other spacing has also been identified [52,53]. We have recently identified a RARE in the hepatic glucokinase gene (*Gck*) promoter, which is involved in the RA-induced *Gck* expression in primary rat hepatocytes [54]. Table 1 shows a summary of RAREs separated by different numbers of nucleotide spacing in representative genes.

**Table 1:** Representative genes bearing retinoic acid response element (RARE) consensus motifs. All RARE sequences are from mouse genome except *Gck*, which is from rat. *Socs3*, the suppressor of cytokine signaling 3; *Msi2*, musashi RNA-binding protein 2; *Crbp2*, cellular retinol binding protein II; *Crabp2*, cellular retinoic acid binding protein II; *Gck*, glucokinase; *Epo*, erythropoietin; *Cyp26a1*, cytochrome P450 26A1; *Rarb2*, retinoic acid receptor β 2; *Rqcd1*, RCD1 required for cell differentiation 1 homolog; *Dedd*, death effector domain containing; *Mafa*, v-maf musculoaponeurotic fibrosarcoma oncogene family, protein A; *Nrp1*, neuropilin 1; *Tr2-11*, testicular receptor 2-11; *Trim16*, tripartite motif-containing protein 16. Nucleotides separating the two motifs are shown in lower-case letters.

Type	Gene	RARE Sequences	References
DR0	*Socs3*	AGTTCA AGGTCA	Moutier *et al.* [48]
	*Msi2*	GGGTCA AGGTCA	
DR1	*Crbp2*	AGGTCA c AGTTCA	Bastien *et al.* [55]
	*Crabp2*	TGACCT c TGCCCT	Durand *et al.* [56]
DR2	*Gck*	TGACCT tg TGACAC	Li *et al.* [54]
	*Epo*	GGGTCA ag AGGTCA	Brade *et al.* [57]
DR5	*Cyp26a1*	AGTTCA cccaa AGTTCA	Loudig *et al.* [58]
	*Rarb2*	GTTCAC cgaaa GTTCAC	Sucov *et al.* [43]
Simple DR8	*Rqcd1*	GGGTCA gaggtgag AGGTCA	Moutier *et al.* [48]
	*Dedd*	AGGTCA cgatctgg AGTTCA	
Composite DR8	*Mafa*	AGGTCA ga AGTTCA AGGTCA	
	*Nrp1*	GGATCA aa AGTTCA AGGTCA	
IR0	*Trim16*	GGGTCA TGACCC	
	*Tr2-11*	GGGTCA CGAACT	Lee and Wei [59]

## 4. Nuclear Receptors (NRs) Mediating Retinoic Acid (RA) Effects

VA is stored mainly in the liver and in small amount in other tissues. The ability of cells to metabolize retinol to RA determines its availability to act in those cells and cells around them if RA can act in a paracrine manner [36]. Thus, VA status of an animal and the developmental, differentiation, and metabolic states of the body’s cells may influence the production and/or delivery of RA at certain stages or loci; which in turn, alter the activation states of NRs at RAREs located in the promoters of specific genes [60].

In addition to RARs and RXRs, other transcriptional factors have been thought to mediate RA signals. RA activates RARs and peroxisome proliferator activated receptor β/δ (PPARβ/δ) [61,62], which associate with RXRs to form heterodimers and act as ligand-regulated transcription factors through binding to RAREs located in the promoters of target genes and modulate their transcription [63,64]. In addition, hepatic nuclear factor 4α (HNF4α) and chicken ovalbumin upstream promoter-transcription factor II (COUP-TFII), may also mediate RA effects [54,65,66].

### 4.1. Introduction of Nuclear Receptors (NRs)

NRs encompass members of a large superfamily of evolutionarily related transcriptional factors that mediate a complex array of extracellular signals into transcriptional responses and therefore control a broad range of biological processes, including development, organ homeostasis, metabolism, immune function, and reproduction [67,68]. There are 48 known NRs in the human genome [69], approximately one-half of which are receptors for endocrine steroids (*i.e.*, corticosteroids, progesterone, androgens, and estrogens), fat-soluble vitamins A and D, thyroid hormone, fatty acids, oxysterols, bile acids, and numerous xenobiotic lipids derived from the diet [67]. The second half of these NRs represents the so-called “orphan” receptors, whose ligands, target genes, and physiological functions were initially unknown [67,70,71,72].

As transcriptional factors, NRs exhibit a specific modular structure including six regions, A, B, C, D, E and F, with different degrees of evolutionary conservation. The variable N-terminal A/B region contains an autonomous transcriptional activation function domain-1 (AF-1). The highly conserved region C that corresponds to the core of the DNA-binding domain (DBD) is responsible for specific binding to the response elements in gene promoters. Region E contains the ligand-binding domain (LBD), a ligand-dependent transcriptional activation function domain-2 (AF-2) and a dimerization interface. A variable region D functions as a flexible hinge between the DBD and LBD and contains the nuclear localization signal. Some but not all NRs contain a variable C-terminal region F of unknown function [70,73,74,75]. NRs can bind directly to DNA as monomers, homodimers, or heterodimers through the DBD [76,77]. Upon ligands binding, most NRs undergo a conformational change that coordinately dissociates co-repressors and facilitates recruitment of co-activators to enable transcriptional activation of target genes [70,78], while others recruit co-repressors and suppress gene expression [79,80].

### 4.2. RARs Are Primary Mediators of RA Signaling

RARs mediate RA-regulated gene expression directly at the transcriptional level [81]. RARs associate with RXRs to form RAR/RXR heterodimers, which regulate transcription following binding to RAREs located in the promoters of a variety of genes and activation by their cognate ligands [63]. In vertebrates, there are generally three RARs that bind to all-*trans* RA and its isomer 9-*cis* RA with high affinity [82,83]. It has been reported that over 500 genes are responsive to either all-*trans* RA or 9-*cis* RA [84]. However, RARs are not only ligand-dependent regulators of transcription but also display extra-nuclear non-transcriptional effects and activate kinase cascades, which are integrated in the nucleus [3]. For example, all-*trans* RA-activated RAR can activate protein kinase C δ in myeloid leukemia and breast cancer cell lines [85] and CK2 in vascular smooth muscle cells [86].

The first RAR, RARα (NR1B1), was cloned by using a consensus oligonucleotide probe corresponding to a highly conserved sequence in the DBD of several members of NR family in 1987 [87]. Shortly after, RARα was cloned independently by another group using a different probe corresponding to a novel sequence similar to the DBD of the steroid hormone receptor [88]. Several years later, two additional RARs, RARβ (NR1B2) and RARγ (NR1B3), were identified [89,90]. These RARs are encoded by distinct genes located in different chromosomes that produce multiple isoforms due to alternative splicing or to the use of different promoters. So far, three [91,92], five [93,94], and two isoforms [95] have been described for RARα, RARβ, and RARγ, respectively.

Comparisons of amino acid sequences of three RARs revealed that the interspecies conservation of a member of the RAR subfamily was much higher than the conservation of all three receptors within a given species [23]. In addition, each of the RARs exhibits a spatially and temporally specific pattern of expression during embryonic development [96], with RARα almost ubiquitously expressed [97,98], and RARβ and RARγ displaying more restricted expression patterns [97,99]. This evidence indicates that RARα, RARβ, and RARγ each may have their own specific functions. It was reported that *Rara* null mice displayed some of the features of VA deficiency (VAD) with decreased viability, growth deficiency, and male sterility, due to degeneration of the seminiferous epithelium as well as other congenital malformations, such as webbed digits [100]. Most of the defects of *Rara* null mice can be reversed by treatment with RA [101]. Mice null for *Rarb* exhibit locomoter defects [102] and a selective loss of striosomal compartmentalization in the rostral striatum [103]. *Rarg* null mice display some defects associated with VAD, which can be rescued with RA treatment, indicating that RARγ mediates some of the retinoid functions *in vivo* [104].

RARs-mediated RA signaling plays critical roles in regulating energy metabolism. In a recent study, Brun *et al.* [105] destroyed RA signaling in pancreatic β cells of adult mice by overexpressing a dominant-negative RARα mutant using an inducible Cre-Lox system under the control of the pancreas duodenal homeobox gene promoter. They found that hypomorphism for RAR in β cells led to an age-dependent decrease in plasma insulin level in the fed state and when challenged with glucose. These animals exhibited decreased β cell mass and impaired glucose-stimulated insulin secretion (GSIS). In isolated islets, mRNA expression of *Gck* and glucose transporter 2 gene (*Glut2*) was suppressed due to the overexpression of dominant-negative RARα mutant [105]. This evidence suggests an essential role of RARs in maintaining pancreatic β cell function and mass.

In cultured adipocytes, activated RAR promotes energy expenditure by up-regulating the expression of genes encoding upcoupling protein 1 (*Ucp1*) and hormone sensitive lipase (*Hsl*) [106]. RAR(s) is also involved in the regulation of adipogenesis. It has been shown that RARγ mediates RA-inhibited adipocyte differentiation by controlling the expression of genes involved in adipogenesis, such as kruppel-like factor 2 (*Klf2*) [107]. In addition, RARs modulate the expression of metabolic genes in murine hepatocytes, such as *Gck*, insulin-like growth factor-binding protein 1 (*Igfbp1*) [54], and the cytosolic form of phosphoenolpyruvate carboxykinase (*Pck1*) [108,109], and thus contribute to energy metabolism.

### 4.3. The Role of RXRs in RA Signaling

In addition to forming heterodimers with RARs, RXRs can associate with RXRs and other NRs to respectively form homodimers and heterodimers. They regulate transcription following binding to RAREs located in the promoters of target genes and responding to the levels of their cognate ligands. In vertebrates, there are generally three RXRs that bind to 9-*cis* RA with high affinity, but not to all-*trans* RA [82,83]. Whereas the physiological role of all-*trans* RA is no longer disputed, the 9-*cis* RA, initially identified as a *bona fide* RXR ligand *in vitro* [100], has not been detected in serum or multiple tissues except pancreas [110,111]. Recently, two natural ligands, docosahexanoic acid [112] and β-apo14′-carotenal [113], were respectively demonstrated to activate and repress RXR transcriptional activity. However, these molecules are not RXR-specific ligands as they can regulate the transcriptional activity of other NRs including PPARs [114].

The first RXR, RXRα (NR2B1), was isolated by using a probe corresponding to the DBD of RARα in a low stringency screen of human liver and kidney cDNA libraries [115]. Subsequently, two additional RXRs, RXRβ (NR2B2) and RXRγ (NR2B3), were isolated [116]. Like RARs, RXRs are encoded by different genes and display distinct pattern of expression. RXRα and RXRβ are widely distributed, whereas abundant expression of RXRγ is limited to only a few tissues [117]. The multiplicity of receptor isotypes and different combinations of RAR/RXR and RXR/RXR within the cell facilitate the mediation of diverse effects of RA. For example, genetic studies have established RXRα/RAR heterodimers as the main functional units transducing RA signals during development, and specific heterodimers (RXRα/RARα, RXRα/RARβ, and RXRα/RARγ) are involved in given developmental processes [100]. Germline mutations in the RXR genes induce either in utero lethality through congenital heart defects (*Rxra*), or metabolic and behavioral defects (*Rxrb*, *Rxrg*) [118]. It has been shown that one copy of *Rxra* allele is sufficient to perform most of the functions of RXRs in mice, indicating that RXRα might be the major player [119].

In addition to RARs, a number of other members of the NR superfamily, can form heterodimeric complexes with RXRs. These include small heterodimer partner (SHP)/NR0B2, liver X receptor α (LXRα)/NR1H3, LXRβ/NR1H2, farnesoid X receptor (FXR)/NR1H4, constitutive androstane receptor (CAR)/NR1I3, thyroid hormone receptor α (TRα)/NR1A1, TRβ/NR1A2, PPARα/NR1C1, PPARβ/δ/NR1C2, PPARγ/NR1C3, vitamin D3 receptor (VDR)/NR1I1, pregnane X receptor (PXR)/NR1I2, photoreceptor cell-specific nuclear receptor (PNR)/NR2E3, ErbA-related protein 2 (EAR2)/NR2F6, nerve growth factor-induced gene B (NUR77)/NR4A1, and nuclear receptor related 1 protein (NURR1)/NR4A2 [73,114,120]. However, the activation state of RXR differs among these heterodimers and seems to depend on the nature of its partner [121]. For instance, in the case of RAR/RXR heterodimer, it is believed that both partners of the heterodimer can be transcriptionally active. However, the ligand-bound RXR is not active unless its RAR partner is itself liganded [122]. In a PPAR/RXR heterodimer, both PPAR and RXR can bind their cognate ligands and activate transcription, with the binding of both ligands resulting in synergistic activation. Heterodimers in this case are referred to as permissive. Similarly, LXR/RXR heterodimer retains 9-*cis* RA responsiveness, further supporting that the view that RXR can be an active partner [71]. In contrast, the TR/RXR and VDR/RXR heterodimers are thought to be non-permissive, as they are activated by the TR ligand triiodothyronine (T3) and VDR ligand 1,25-dihydroxy-VD3 (calcitriol), respectively, but not by RXR-specific ligands. It is generally believed that in a non-permissive heterodimer, RXR is incapable of ligand binding and thus is often referred to as a silent partner. However, recent data indicated that RXR was able to bind ligand and lead to dissociation of co-repressors from TR, thus modulating heterodimer activity [123].

As obligate heterodimeric partners for the abovementioned NRs, RXRs can integrate a number of metabolic signaling pathways and therefore contribute to energy metabolism. Administration of RXR-specific agonist LG100268 to diabetic rats leads to increased expression of metabolic genes, such as carnitine palmitoyltransferase I (*Cpt1*), stearoyl-CoA desaturase 1 (*Scd1*), and fatty acid translocase (*Cd36*) in the liver [124]. In addition, the synergistic effect of retinoids and insulin on the transcription of gene encoding sterol regulatory element-binding protein 1 (*Srebp-1c*) is mediated by RXR in primary rat hepatocytes [125]. In cultured preadipocytes, RXR-selective agonists induce adipose differentiation [126]. They also affect the expression of genes encoding key regulatory enzymes in adipose tissue. It has been shown that LG100268 increases the transcription of gene encoding tumor necrosis factor α (*Tnfa*) and suppresses the transcription of *Cpt1* and *Cd36* in adipose tissue of Zucker fatty rats [124]. In skeletal muscle, LG100268 increases the expression of *Scd1* and *Cd36* [124]. For specific RXR isotypes, it has been shown that hepatocyte RXRα-deficient mice have reduced food intake, increased body weight, and improved glucose tolerance when compared with wild type control mice. This is possibly due to the compromise of PPARα signaling pathway and increased serum IGF-1 level in the mutant mice [127]. In addition, the selective deletion of RXRα in adipocytes renders mice resistant to chemical- and high fat diet (HFD)-induced obesity [128], indicating a role of RXRα in lipogenesis. For GSIS, it has been shown that co-overexpression of RXRα and PPARα and co-overexpression of RXRα and PPARγ potentiate and attenuate GSIS in INS-1 cells, respectively [129]. Suppression of RXRβ in both pancreatic β cells of transgenic mice and MIN6 cells enhances GSIS [130].

### 4.4. HNF4α and Its Potential Association with RA Signaling Pathway

HNF4 represents a subclass of transcriptional factors in the NR superfamily. It was originally detected in crude liver extracts as protein binding to a DNA element required for the transcription of the TTR gene in hepatoma cells in 1989 [131]. Then, HNF4 was purified from rat liver nuclear extracts using DNA affinity column chromatography. HNF4 cDNA clone was then isolated using synthetic oligonucleotides derived from amino acid sequence of the purified HNF4 [132]. Three different genes coding for three different isotypes of HNF4 have been identified, HNF4α and HNF4γ in mammals, drosophila, and xenopus, and HNF4β in xenopus [133].

HNF4α gene (*Nr2a1*) covers ~30 kb and contains 13 exons; exons 2–10 and the alternatively spliced exons 1a, 1b, 1c and 1d [134]. *Nr2a1* encodes a total of nine isoforms with various 3′ truncations and its expression is driven by two distinct promoters that are physically separated by more than 45 kb [135], the P1 promoter that drives expression of splice variants HNF4α1-6 in the liver, kidney, and intestine/colon and the P2 promoter that drives expression of splice variants HNF4α7-9 in the intestine/colon, stomach, and β cells of the pancreas [136]. Whereas HNF4α1-6 includes exons 1a and 2–10 (HNF4α1-3) or exons 1a, 1b, and 2–10 (HNF4α4-6), HNF4α7-9 includes exons 1d and 2 [134]. The expression of HNF4α variants also varies with development and differentiation [137].

HNF4α contains a zinc finger region and binds DNA as a dimer. The C-terminal contains a large hydrophobic domain reminiscent of the dimerization and LBD of other members [138]. Although HNF4α displays significant sequence similarities to the mammalian RXRα, it does not heterodimerize with any of the other NRs identified [139]. Instead, HNF4α binds as a homodimer to a relatively degenerate consensus DNA sequence consisting of two DR elements separated by one or two nucleotides and generally acts as a positive transcriptional regulator [140]. Serine/threonine phosphorylation may play a role in the DNA-binding activity of HNF4α [141]. Animal studies have shown that *in vivo* phosphorylation of HNF4α depends on the diets; it is decreased by a carbohydrate-rich diet and is increased by fasting or in refed animals given glucagon or isoproterenol and phosphodiesterase inhibitors [142].

HNF4α plays important roles in embryonic development and adult physiology. In mice, expression of HNF4α is observed in the primary endoderm as early as E4.5 and continues in extra-embryonic visceral endoderm of the yolk sac throughout development. Its expression in the development fetus is first detected in the liver bud and gut at E8.5 and subsequently in the developing pancreas and kidney by E10 [143,144]. Disruption of the murine *Nr2a1* leads to embryonic lethality due to a dysfunction of the visceral endoderm [145,146]. In adults, HNF4α is a central regulator of gene expression in cells playing critical roles in metabolic homeostasis, including hepatocytes, enterocytes, and pancreatic β cells [147]. Liver-specific knockout of *Nr2a1* in mouse results in accumulation of lipid in the liver, reduction of serum cholesterol and triglyceride levels, and increase of serum bile acid concentrations [148]. It was reported that drosophila HNF4 (dHNF4) regulates lipid mobilization and β-oxidation [149]. dHNF4 null mutant larvae are unable to efficiently mobilize stored fat during starvation due to reduced expression of genes that control lipid catabolism and β-oxidation. Recent studies have shown that HNF4α can activate hepatic *Gck* expression by binding to its promoter [150,151,152]. Pancreatic β cell-specific *Nr2a1* knockout mice have impaired GSIS [153]. *In vitro* studies have shown that HNF4α is important for the development and functions of pancreatic β cells. For example, HNF4α activates the insulin gene expression through indirect and direct mechanisms [154]. HNF4α also regulates the expression of other pancreatic β cell genes implicated in glucose metabolism and nutrient-induced insulin secretion, including *Glut2* and *Lpk* [155].

In human, heterozygous mutations in *Nr2a1* are associated with maturity-onset diabetes of the young type 1 (MODY1), an autosomal dominant genetic disorder that is characterized by early onset type 2 diabetes. The first HNF4α mutation was found to be a nonsense mutation in codon 268, Q268X [156]. This mutation generates a truncated protein that contains an intact DBD but lacks part of the AF-2 region. Functional studies of this mutation have shown that the cause of diabetes is a loss-of-function mutation rather than a dominant-negative or gain-of-function mechanism [138]. Eight additional HNF4α mutations have been associated with type 2 diabetes: F75fsdelT [157], K99fsdelAA [158], R154X [159], G115S [160], R127W [161], V255M [162], E276Q [163], and V393I [164]. In addition, genetic studies have shown that single nucleotide polymorphisms in the P2 promoter of *Nr2a1* are associated with type 2 diabetes in some populations [165,166,167]. No such mutations have been identified in the P1 promoter [136]. Therefore, not only do *Nr2a1* mutations cause MODY, but variations of *Nr2a1* are associated with a genetic predisposition to type 2 diabetes [153].

HNF4α, although initially believed to be an orphan NR, its activity can be modulated by fatty acyl-coenzyme A (CoA), which may act as agonistic or antagonistic ligands depending on chain length and degree of saturation [168], and also by protein kinase A-mediated phosphorylation [142]. X-ray crystal structure together with gas chromatography/mass spectrometry analyses of HNF4α LBD reveals that C14–C18 fatty acids almost fills in a well-defined hydrophobic pocket, suggesting that they might be natural ligands for HNF4α [169,170]. However, these fatty acids are tightly bound and could not be displaced from the receptor without denaturing the protein [169]. Mutations of HNF4α LBD that disrupt fatty acid binding decrease HNF4α transcriptional activity [169]. Consistently, a recent study showed that fatty acids released from triglycerides can activate HNF4 in fasted drosophila, which in turn may drive fatty acid oxidation [149].

Although binding to its cognate DNA binding site as homodimer, HNF4α can interact physically and functionally with HNF1α to regulate transcription of target genes. HNF4α and HNF1α bind directly to one another and this cooperative interaction can occur on target genes with binding sites for both factors in their regulatory sequences [171,172]. A second type of protein-protein interaction is a single-site tethering interaction, where one factor binds to the DNA and the second factor binds to the first factor to affect regulation [173]. HNF1α binds to HNF4α through interaction with HNF4α residues 337–368, while HNF4α binds to HNF1α through interaction with HNF1α residues 280–440 [174]. For example, the apolipoprotein C-III gene (*Apoc3*) promoter contains a HNF4 but not HNF1 binding site, and HNF1α suppresses HNF4α-activated *Apoc3* expression and decreases HNF4α promoter occupancy [175]. Another transcriptional factor that can functionally interact with HNF4α is COUP-TFII. Studies have shown that HNF4α interacts with COUP-TFII and exerts a synergistic effect on activating *Hnf1a* [176] and *Pck1* [177] promoters and that HNF4α and COUP-TFII interact to modulate the transcription of cholesterol 7α hydroxylase gene (*Cyp7a1*) [178]. On the other hand, COUP-TFII negatively affects gene transcription by interfering with HNF4 on the promoters of apolipoprotein genes *Apoa1* [179], *Apoa2*, *Apob*, and *Apoc3* [180,181]. It is believed that HNF4 and COUP-TFII interact through the LBD of HNF4 [182].

A relationship between retinoids and HNF4 has been indicated in the regulation of the hepatocyte phenotype. It was found that RA-mediated down-regulation of α-Fetoprotein gene was dependent on RA-mediated inhibition of HNF1 and HNF4 expression in Hep3B cells [183]. Since no RARE has been found in the HNF4 gene promoter, the mechanism for RA-mediated inhibition of HNF4 remains to be investigated. One possibility is that RA treatment results in reduced expression or activity of RARs or RXRs, which changes the interaction of HNF4 and RXR homo- and heterodimers at cognate binding site and contributes to the effect [183]. On the other hand, overexpression of HNF4α attenuates the RA-induced *Gck* expression in primary rat hepatocytes [54]. And HNF4α regulates the intracellular transport of RA by activating the transcription of *Crbp2* [184]. Furthermore, it is suggested that HNF4 and RXRα compete for occupancy at the same DR2 element in cytokine erythropoietin gene (*Epo*) promoter and sequentially regulating its expression during embryogenesis [185]. During the E9.5–E11.5 phase of mouse fetal liver, expression of *Epo* is regulated by RAR/RXRα. From E11.5 onward, *Epo* expression is mainly regulated by HNF4 [185].

### 4.5. The Role of COUP-TFII in RA Signaling

COUP-TFs are among the most extensively studied orphan NRs with no identified physiological ligand. COUP-TF was first identified as a homodimer that binds to a DR element in the chicken ovalbumin promoter [186,187]. In humans, COUP-TFs consist of at least three members, COUP-TFI (also called NR2F1 or EAR3), COUP-TFII (also called NR2F2 or apolipoprotein-AI regulatory protein-1 [ARP1]), and the more distant COUP-TFIII (also called NR2F6 or EAR2) [188]. COUP-TFs are the most evolutionarily conserved NRs among all species, with the LBDs of COUP-TFI or II being essentially identical in vertebrates [66]. Deletion of either COUP-TFI gene (*Nr2f1*) or COUP-TFII gene (*Nr2f2*) in mice is lethal [189]. However, COUP-TFII is required earlier in development than COUP-TFI [190].

COUP-TFII was first purified from HeLa cell nuclear extracts by DNA affinity column chromatography and EMSA assays [191]. It was then cloned based on its high homology to COUP-TFI [191,192]. *Nr2f2* is located on chromosome 15q26 and widely expressed in multiple tissues and organs throughout embryonic development [193]. COUP-TFII homozygous knockout (*Nr2f2^−/−^*) mice exhibit defects in angiogenesis and heart development and die before E10.5 [194], and the female heterozygous knockout (*Nr2f2^+/−^*) mice have impaired reproductive function, whereas the male *Nr2f2^+/−^* mice are normal and fertile [195].

Although the expression level of COUP-TFII is dramatically reduced in adults [196], COUP-TFII functions as an important regulator of metabolic homeostasis [197]. For the pancreas, conditional inactivation of *Nr2f2* in mouse pancreatic β cells leads to impaired glucose sensitivity, abnormal insulin secretion, and insulin resistance [198], which are also observed in mice with deletion of *Nr2a1* in pancreatic β cells [153,199,200]. *In vitro* studies have shown that siRNA-mediated knockdown of *Nr2f2* strongly increased insulin gene transcription and content in 832/13 INS-1 cells and pancreatic islets, whereas adenovirus-mediated overexpression of *Nr2f2* significantly reduced insulin gene transcription and content in these cells [201], indicating a critical role of COUP-TFII in controlling the biosynthesis and secretion of insulin.

For the adipose tissue, *Nr2f2^+/−^* mice display reduced adiposity and expression levels of key genes for adipogenesis. In addition, these animals have improved glucose homeostasis and increased energy expenditure, and are protected from HFD-induced obesity and insulin resistance [202]. In keeping with this, it was shown that knockdown and overexpression of *Nr2f2* in 3T3-L1 preadipocytes enhanced and suppressed adipogenesis, respectively [203].

For skeletal muscle cells, COUP-TFII modulates genes and pathways involved in glucose and lipid utilization. In the cultured C2C12 skeletal muscle cell model, siRNA-mediated knockdown of *Nr2f2* expression significantly reduced expression of metabolic genes such as *Nr1c1*, *Fabp3*, *Cpt1*, *Ucp1*, ATP-binding cassette subfamily A member 1 (*Abca1*), and ATP-binding cassette subfamily G member 1 (*Abcg1*) [204]. In the same cell line, overexpression of *Nr2f2* directly decreases the expression of *Glut4* [205].

For the liver, it has been shown that COUP-TFII synergizes with HNF4 to induce the transcription of *Cyp7a1* in human hepatoma cell line HepG2 [178]. Our recent studies indicate that COUP-TFII influences RA- and insulin-mediated expression of metabolic genes such as *Gck* and *Pck1* in primary rat hepatocytes [54].

Notably, the expression of COUP-TFII is modulated by the nutritional status. *Nr2f2* mRNA levels are increased in the pancreas and liver of fasted mice and decreased upon feeding with a carbohydrate-rich diet [201]. Given the critical role of COUP-TFII in adipogenesis, it is therefore speculated that down-regulation of COUP-TFII by over nutrition may underlie the development of obesity [206].

Despite that COUP-TFII can activate transcription in certain cell types and promoter contexts, it acts primarily as repressor of ligand-mediated NR signaling pathways via both protein-protein and protein-DNA interactions. For example, COUP-TFII can bind DNA by a Zinc finger DBD on a variety of cognate binding sites, which contain different imperfect DR or IR elements [207]. These binding sites can also be competitively recognized by other NRs. For instance, HNF4α response elements are bound by COUP-TFII on most promoters [208]. Although COUP-TFII primarily forms a homodimer, it can also form heterodimer with RXR and act as a repressor for gene expression [209]. In addition, COUP-TFII can directly influence basal or activated transcription by protein-protein interaction with transcription factor II B [210] or other transcription factors [211]. A COUP-TFII interacting protein, p62, possibly plays a role in mediating the differential regulation of gene expression by COUP-TFII [212].

High expression of COUP-TFII is induced preferentially by all-*trans* and 9-*cis* RA during retinoid-induced differentiation of P19EC cells [213]. This effect is possibly mediated by RARs or RXRs [214]. RA enhances the COUP-TFII occupancy at *Rarb* promoter and leads to increased expression of RARβ target genes in breast cancer cells [215]. In addition, the pulmonary COUP-TFII expression is up-regulated after prenatal treatment with RA in a nitrofen model of congenital diaphragmatic hernia [216]. In zebrafish, RA modulates COUP-TFII expression during embryogenesis [217].

On the other hand, it was shown that COUP-TFs were involved in the modulation of RAR- and RXR-mediated responses to retinoids during embryogenesis [188]. COUP-TFII inhibits RA actions in the mouse fetal lung at the pseudoglandular phase of lung development [218]. We have shown that overexpression of COUP-TFII affects RA-mediated *Gck* expression in primary rat hepatocytes [54]. Furthermore, it has been shown that COUP-TFII plays a role in mediating the endothelial trans-differentiating properties of RA in breast cancer cells [219].

It seems that COUP-TFII and RA affect one another’s function in certain physiological processes. Recently, COUP-TFII was identified as a low-affinity RA receptor, and RA was able to promote COUP-TFII to recruit co-activators and activate COUP-TFII reporter construct [66]. Due to the low RA endogenous levels in physiological environment, whether RA is the physiologically relevant ligand for COUP-TFII remains to be determined [66]. Nevertheless, these observations suggest a linkage between RA and COUP-TFII signaling pathways.

### 4.6. The Role of PPARβ/δ in RA Signaling

PPARs are ligand-activated transcriptional factors that belong to the NR superfamily. The family of PPARs comprises three members: PPARα (NR1C1), PPARβ/δ (NR1C2), and PPARγ (NR1C3) [220], each of which is encoded by a distinctive gene, e.g., human PPARα, β/δ, and γ are encoded by separate genes located on chromosome 22 [221], 6 [222], and 3 [223], respectively. The three PPAR members share a high degree of homology but differ in tissue distribution, ligand specificity, and physiological roles [224]. PPARα is highly expressed in tissues with elevated fatty acid catabolism such as liver, intestine, brown adipose tissue, and heart [225]. PPARγ has two forms, PPARγ1 that is expressed in a variety of cell types and organs such as immune cells, gut, brain, and vascular cells [226], and PPARγ2 that is almost exclusively expressed in adipose tissue [227]. PPARβ/δ expression is very high in the small intestine and keratinocytes; high in liver, colon, kidney, and skin; and low in other tissues including brain, heart, lung, skeletal muscle, testis, spleen, and thymus [220]. It is of importance to note that expression levels of PPARs are subject to regulation by diets and nutrient status in a tissue-dependent manner. For instance, PPARβ/δ expression is dramatically decreased in liver [228] and increased in skeletal muscle after an overnight fasting [229].

PPARβ/δ plays a critical role in regulating lipid metabolism and insulin sensitivity. It has been shown that targeted activation of PPARβ/δ in adipose tissue induces expression of genes required for fatty acid oxidation and energy dissipation, which in turn leads to improved lipid profiles and reduced adiposity in mice. In contrast, mice null for *Nr1c2* challenged with a HFD have reduced energy uncoupling and are prone to obesity [230]. In the skeletal muscle, PPARβ/δ induces the expression of genes involved in fatty acid oxidation [230,231,232]. Given that PPARβ/δ is the most abundant isoform among the three PPAR members in the skeletal muscle, PPARβ/δ-induced fatty acid oxidation might be critical for the overall improvement of lipid profile and insulin sensitivity. In keeping with this, it was shown that PPARβ/δ agonists improved glucose tolerance and insulin sensitivity in different mouse models of obesity [233].

It is thought that PPARβ/δ regulates the expression of genes implicated in the many physiological processes by three mechanisms: direct induction, indirect regulation of transcription, and non-genomic action. Directly, PPARβ/δ forms heterodimers with RXRs and binds to PPAR responsive element (PPRE) located in the promoter of target genes upon ligand binding [234]. A PPRE consists of an imperfect DR1 of AGGTCA [235]. Functional PPREs have been identified in a large number of genes involved in nutrient metabolism. Activation of the PPARβ/δ/RXR heterodimer is induced by either PPAR ligands or RXR ligands, while co-existence of PPAR ligands and RXR ligands results in synergistic induction of target genes [236]. Indirectly, unbound PPARβ/δ physically interacts with other transcription factors, such as the co-repressor B cell lymphoma-6 (BCL6), and prevents the anti-proliferative effects of BCL6 activation [237,238]. Activation of PPARβ/δ leads to release of BCL6, which in turn exerts its function. In addition, PPARβ/δ can indirectly affect the transcription of genes involved in nutrient metabolism by inhibiting nuclear factor κ B (NF-κB) [220]. For the non-genomic action, it has been shown that the activated PPARβ/δ can bind directly to phosphokinase Cα in platelets to inhibit adhesion [239].

Two proteins belonging to the intracellular lipid binding proteins (iLBP) family, CRABP-II and FABP5, respectively deliver RA from sites of synthesis in the cytosol to RARs and PPARβ/δ in the nucleus. In doing so, CRABP-II and FABP5 significantly sensitize the cells to RA biological activities [240]. *In vitro* studies have shown that RA binds to PPARβ/δ with high affinity and efficiently activates PPARβ/δ-mediated transcription [241]. In certain breast cancer cell lines, curcumin suppresses the expression of FABP5 and PPARβ/δ and therefore inhibits the expression of PPARβ/δ target genes in response to RA [242].

On the other hand, CRABP-II transports RA to RAR that mediates the “traditional” activities of RA as mentioned above [243,244]. The differential cellular response to RA seems to be determined by the relative expression of CRABP-II and FABP5 in specific cells. RA controls expression of RAR and PPARβ/δ target genes in cells with high and low CRABP-II/FABP5 ratio, respectively [42]. It is of interest to note that both RA pathways could be involved in the same physiological process. For instances, RA-induced neuronal differentiation is mediated through RAR in the early stages and through PPARβ/δ in the late stages [245]. In cultured adipocytes, RA promotes energy expenditure partially by up-regulating the expression of genes that are jointly controlled by RAR and PPARβ/δ [106].

### 4.7. Promiscuous Binding of RARs, RXRs, HNF4, and COUP-TFII to RAREs

RARs, RXRs, HNF4, COUP-TFII, and other transcriptional factors can bind to a common DNA binding site (RARE), especially DR1 element, in a number of gene promoters and regulate their activity. For example, except for the abovementioned gene promoters, the hepatitis B virus enhancer 1 (*Hbve1*) and *Hnf1b* promoter contain DR1 element that can interact with HNF4, RXRα, or COUP-TF [246,247]. HNF4 and PPARα, respectively activate and suppress *Apoc3* and transferrin promoter activity, through binding to the same DR1 element [248,249]. HNF4, COUP-TFII, and PPARα regulate human PPARα gene promoter activity through binding to the same DR1 site [250]. In addition, human *Apoa1* hepatic enhancer contains three regulatory regions, two of which are common binding sites for HNF4α, PPARα, RXRα, COUP-TFII, and LXRs [251]. In the case of murine *Crbp2*, an HNF4/COUP-TFII response element was shown to function as a promiscuous binding site for RXR [184]. Our recent studies also indicate that the RARE of hepatic *Gck* promoter can interact with RARα, HNF4α, and COUP-TFII and affect RA- and insulin-induced *Gck* expression in primary rat hepatocytes [54]. For the three RAREs located in the *Pck1* promoter, HNF4α, RARα, RXRα, PPARα, and COUP-TFII bind to RARE1/RARE2, whereas PPARα and RXRα bind to RARE3 *in vivo* [108]. Importantly, binding of certain NR(s) is subject to modulation by VA status of the animal. For instance, PPARα binding at either RARE1/RARE2 or RARE3 in the liver of VAD mice is reduced when compared with that in the liver of VAS mice [108].

## 5. Conclusions and Future Perspectives

Elucidating the molecular mechanisms underlying the diverse effects of RA is a challenging problem in hormone signaling [63]. The NRs summarized above regulate RA-responsive genes at the transcriptional level by interacting with RAREs in their promoters or enhancers, which appear to be highly promiscuous. RARs, RXRs, HNF4α, COUP-TFII, and PPARβ/δ bind to a given RARE as homodimers or heterodimers and their complex interaction can lead to significant differences in the expression of their target genes in different tissues. In addition, the co-existence of these NRs in certain cell types suggests that their relative expression levels and affinity for RARE, as well as protein-protein interactions with other factors binding to proximal and distal regulatory elements are likely to contribute to the diverse RA effects on transcription we have observed (Figure 1). Indeed, our recent studies have shown that adenovirus-mediated overexpression of RARα, HNF4α, and COUP-TFII in primary rat hepatocytes results in dramatic increase in their occupancy at the hepatic *Gck* promoter and in changes in *Gck* expression [54]. Sequence analysis of the promoter reveals the existence of a DR2 element that overlaps with a previously identified HNF4α binding site [151]. However, overexpression of RXRα results in little change in its occupancy at the *Gck* promoter, possibly due to the redundancy of RXRα in hepatocytes or to the nature that RXR/RXR homodimers prefer to bind to DR1 element rather than DR2 element. Interestingly, disruption of nucleotides surrounding the identified RARE also affects the activation of promoter by NRs, raising the question that whether any unknown mediators bound to the promoter region proximal to the RARE play a role. In addition, the precise contribution that each NR makes to the expression of a specific gene deserves to be further investigated.

Liver is a main storage site and an active organ for metabolism of VA. The homeostasis of VA must be maintained to meet optimal physiological requirements. Fluctuation of intracellular RA levels can induce dynamic changes in the binding of the abovementioned NRs to the promoters. This, together with the fact that diets, nutrient status, and metabolic states such as fasting and refeeding modulate the expression levels of certain NRs (e.g., the level of HNF4α expression is high during fasting, decreases in the liver [252] and in the pancreas upon refeeding, and correlates well with the variations in COUP-TFII transcript and protein levels [208]) evokes the question that what are the effects of other nutritional and hormonal stimuli on the promoter context. For example, rats fed a VA deficient diet have lower level of insulin and hepatic *Gck* expression as compared with rats fed a VA sufficient diet [253]. Does insulin together with RA induce a dynamic change in the binding of these NRs to the hepatic *Gck* promoter? Previously, we have shown that RA and insulin converge at the same site of *Srebp-1c* promoter and result in a synergistic induction of mRNA expression of *Srebp-1c* [125]. Whether there is an insulin response element (IRE) located in the hepatic *Gck* promoter? Unfortunately, new strategies seem to be needed for the identification of IRE in the *Gck* promoter as *in vitro* reporter gene construct transfection studies have failed [54,254]. Could there be any interaction between the transcriptional factors bound to the RARE and those bound to the IRE? Insights into the mechanisms underlying the synergistic effect between RA and insulin could serve as a paradigm for understanding how RARs, RXRs, HNF4α, and COUP-TFII determine the response of cells to RA and other hormonal and nutritional signals.

**Figure 2:**
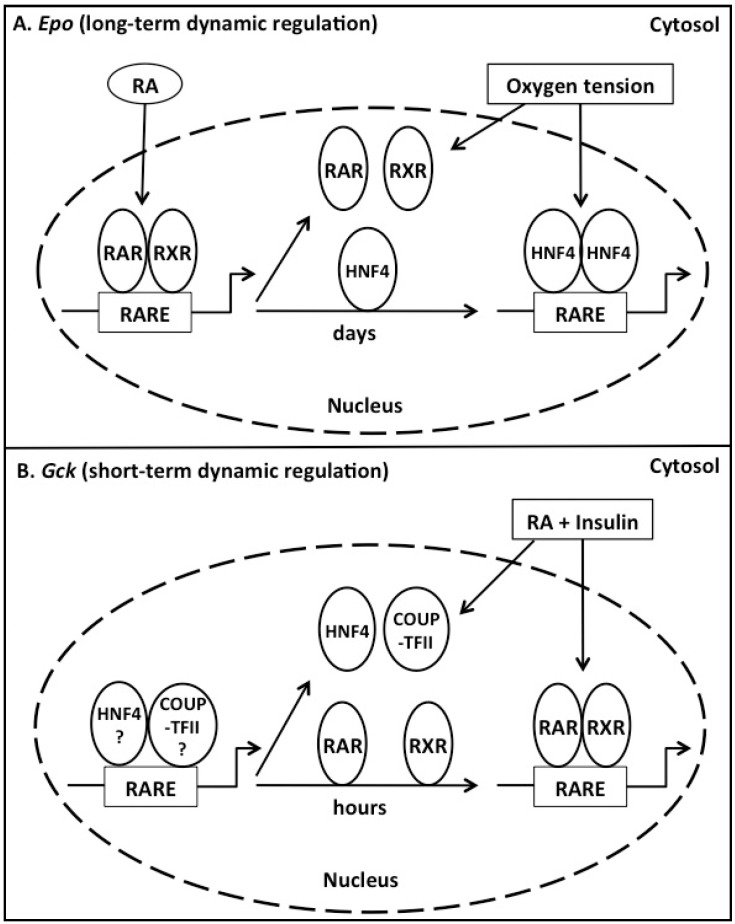
Proposed models of short-term and long-term dynamic regulations of RA-responsive genes. (**A**) Long-term dynamic regulation may take days. For the gene encoding erythropoietin (*Epo*), its transcription is regulated during the E9.5–E11.5 phase of fetal liver erythropoiesis by RAR/RXR heterodimer following binding to RARE located in the promoter and activation by RA. During this phase, other NRs including HNF4, may constantly compete with RAR/RXR for binding to the RARE. Starting from E11.5, RARs and RXRs dissociate from RARE and HNF4/HNF4 homodimer predominantly binds to the RARE and controls *Epo* transcription in response to the environmental stimulus oxygen tension; and (**B**) Short-term dynamic regulation occurs within hours. In the case of hepatic *Gck*, HNF4/HNF4 and COUP-TFII/COUP-TFII homodimers, or HNF4/COUP-TFII and COUP-TFII/RXR heterodimers (as indicated by “?”) bind to RARE in the promoter and control the basal transcription of hepatic *Gck*. Environmental change, such as nutrient influxes, may result in alteration of nutritional (e.g., RA) and hormonal (e.g., insulin) statuses within hours. RA and insulin may modulate the binding of different NRs to the RARE and lead to the replacement of bound dimers with RAR/RXR heterodimer, which in turn, may promote the transcription of hepatic *Gck*.

Dietary nutrients enter the body after ingestion and digestion. This is followed by the fluxes of nutrients into the cells in concert with changes of hormone levels. The rise and fall of nutrient and hormone levels in the blood circulation leads to changes in cellular and whole body metabolic states. This cycle repeats daily and the metabolic changes can be partially attributed to changes of expression levels of metabolic genes in response to both nutritional and hormonal signals. Therefore, for a particular element of a gene’s promoter, the regulation is a dynamic process, which may be mediated by one or more transcriptional factors. Analogously, for a particular RARE of a given gene (e.g., the hepatic *Gck*) in a cell at certain moment in the dynamic regulation process, multiple transcriptional factors may interact with this RARE for the regulation in the next moment or period of time due to the changes of nutritional and hormonal status. As shown in Figure 2, if we use the hepatic *Gck* expression as the example, factors that can determine the association of transcriptional factors with *Gck* RARE and their activities may include RA production rate, expression levels of transcriptional factor (RARs, RXRs, HNF4α, and COUP-TFII), their relative binding affinities and activation states of transcriptional factors in the presence or absence of insulin stimulation. The association or activation of one of these transcriptional factors at the hepatic *Gck* depends on the RA level and strength of insulin stimulation. The RA level may be determined by the dietary VA status [4], whereas the insulin stimulation strength may be controlled by the amount of carbohydrate, fat, and protein ingested in a meal. The sequential regulation of *Epo* by RAR/RXR and HNF4α during embryogenesis certainly demonstrates that this scenario occurs over a course of days [185]. Our observation that retinoids synergize with insulin to induce *Gck* expression in primary rat hepatocytes [255] shows that this dynamic regulation happens in hours. The identification of the hepatic *Gck* RARE and demonstration of its interaction with RARs, RXRs, HNF4α, and COUP-TFII [54] clearly suggest that these four transcriptional factors may contribute to the short-term dynamic regulation of the hepatic *Gck* expression.

Here, we have summarized the VA metabolism and NRs that mediate RA effects. In addition, we provide scenarios that multiple transcriptional factors work together to contribute to the dynamic regulation of a RA-responsive gene. It seems that a RARE can be a convergent point for multiple physiologically relevant signals. Our understanding of transcriptional factors interacting with a RARE seems to be incomplete. Therefore, further studies will reveal more physiologically meaningful mechanisms mediating RA signals.
